# A recombinant avian paramyxovirus serotype 3 expressing the hemagglutinin protein protects chickens against H5N1 highly pathogenic avian influenza virus challenge

**DOI:** 10.1038/s41598-020-59124-x

**Published:** 2020-02-10

**Authors:** Edris Shirvani, Berin P. Varghese, Anandan Paldurai, Siba K. Samal

**Affiliations:** Virginia-Maryland College of Veterinary Medicine, University of Maryland, College Park, MD USA

**Keywords:** Influenza virus, Live attenuated vaccines

## Abstract

Highly pathogenic avian influenza (HPAI) is a devastating disease of poultry and a serious threat to public health. Vaccination with inactivated virus vaccines has been applied for several years as one of the major policies to control highly pathogenic avian influenza virus (HPAIV) infections in chickens. Viral-vectored HA protein vaccines are a desirable alternative for inactivated vaccines. However, each viral vector possesses its own advantages and disadvantages for the development of a HA-based vaccine against HPAIV. Recombinant Newcastle disease virus (rNDV) strain LaSota expressing HA protein vaccine has shown promising results against HPAIV; however, its replication is restricted only to the respiratory tract. Therefore, we thought to evaluate avian paramyxovirus serotype 3 (APMV-3) strain Netherlands as a safe vaccine vector against HPAIV, which has high efficiency replication in a greater range of host organs. In this study, we generated rAPMV-3 expressing the HA protein of H5N1 HPAIV using reverse genetics and evaluated the induction of neutralizing antibodies and protection by rAPMV3 and rNDV expressing the HA protein against HPAIV challenge in chickens. Our results showed that immunization of chickens with rAPMV-3 or rNDV expressing HA protein provided complete protection against HPAIV challenge. However, immunization of chickens with rAPMV-3 expressing HA protein induced higher level of neutralizing antibodies compared to that of rNDV expressing HA protein. These results suggest that a rAPMV-3 expressing HA protein might be a better vaccine for mass-vaccination of commercial chickens in field conditions.

## Introduction

Avian influenza viruses (AIVs) are members of the genus *Alphainfluenzavirus* in the family *Orthomyxoviridae*^1^. They can cause a wide range of clinical disease in chickens. *Alphainfluenzaviruses* are classified into combinations of 18 H (H1-H18) and 11 N (N1-N11) subtypes, based on antigenic differences of their hemagglutinin (HA) and neuraminidase (NA) surface glycoproteins. H1-H16 and N1-N9 subtypes have been found in avian species^2^. AIVs are divided into low pathogenic avian influenza viruses (LPAIVs) that cause restricted respiratory and/or intestinal disease without mortality in specific pathogen free (SPF) chickens, and highly pathogenic avian influenza viruses (HPAIVs) that cause high mortality following an acute systemic infection in SPF chickens^3,4^. Avian influenza is a devastating disease of poultry and a serious threat to public health, which is caused by H5 or H7 subtype of HPAIV^5,6^.

Nationwide vaccination with inactivated virus vaccines and/or viral vectored HA-based vaccines is one of the major policies that is currently implemented against HPAIV in chickens in many countries^7^. Although the greater part of currently used vaccines are inactivated vaccines, this type of vaccine is not the best choice to combat HPAIV infection in poultry. Because not only the effectiveness of these vaccines is suboptimal, but also the processes of production and administration of these vaccines are expensive, time consuming and labor intensive. Furthermore, the inability to easily serologically differentiate infected birds from vaccinated ones is a concern of vaccination with inactivated vaccines containing virus particles^8,9^. On the contrary, viral-vectored HA protein vaccines are a desirable alternative for inactivated vaccines^10–16^. However, the advantages and disadvantages of each virus should be considered when it is chosen as a vector for the development of a HA-based vaccine against HPAIV^17^.Among several viral-vectored HA protein vaccine candidates that have been extensively studied in chickens, recombinant Newcastle disease virus (rNDV) expressing HA protein of HPAIV has shown highly promising results and has been licensed to use in the field^3,10,17–23^.

NDV is a member of genus *Orthoavulavirus* in the family *Paramyxoviridae*^1^. NDV strains are classified into three pathotypes based on the severity of disease which they cause in chickens: lentogenic (low virulent), mesogenic (moderately virulent) and velogenic (highly virulent). The major viral factor that determines the pathotypes of NDV is the fusion protein cleavage site (FPCS) sequence. Cleavage of precursor F0 to F1 and F2 subunits is a prerequisite for paramyxovirus infectivity, tissue tropism and pathogenicity^24,25^. Lentogenic NDV strains contain a mono or dibasic amino acid within the FPCS such that the F0 protein can be cleaved into F1 and F2 subunits only by a trypsin-like protease that is present extracellularly in the respiratory and intestinal tracts. In contrast, mesogenic and velogenic NDV strains have multibasic amino acids at the FPCS that can be cleaved intracellularly by the ubiquitous furin-like protease, resulting in systemic infection^26^. Although both lentogenic and mesogenic NDV strains have been evaluated as human and veterinary vaccine vectors, the mesogenic NDV strain Beaudette C was found to be highly effective because of its systemic replication^27^. However, mesogenic NDV strains are Select Agents and therefore cannot be used as vaccine vectors. Hence, we thought to evaluate another avian paramyxovirus (APMV) serotype as a vaccine vector against HPAIV, which has high efficiency replication in a greater range of host organs, in comparison to NDV strain LaSota, which is restricted only to the respiratory tract^28,29^.

All strains of NDV belong to APMV-1, but officially 20 additional serotypes have been identified^1^. Of these serotypes, APMV-3 strain Netherlands, which belongs to the genus *Paraavulavirus*, has a multibasic FPCS, allowing it to replicate in a wide range of host tissue^30,31^. APMV-3 is highly safe in chickens and turkeys and it is not a Select Agent. Therefore, it can be used as a vaccine vector in chickens and turkeys. Furthermore, APMV-3 is an antigenically distinct APMV serotype; therefore, it can circumvent pre-existing immunity to NDV better than rNDV vector. In this study, we generated a recombinant APMV-3 (rAPMV3) strain Netherlands expressing the HA protein of H5N1 HPAIV using reverse genetics. We evaluated the induction of neutralizing antibodies and protection by rAPMV3 strain Netherlands and rNDV strain LaSota expressing the HA protein against HPAIV and highly virulent NDV challenges in chickens. Our results showed that compared to rNDV expressing HA protein, immunization of chickens with rAPMV-3 expressing HA protein induced higher level of neutralizing antibodies against HPAIV. Moreover, a prime vaccination of broilers with rAPMV-3 expressing HA protein followed by a booster immunization with rNDV expressing HA protein induced even high level of humoral antibody response against HPAIV. Both rAPMV-3 expressing HA protein and rNDV expressing HA protein provided protection in chickens from death following HPAIV challenge. Although both vectors expressing the HA protein induced protective immunity against HPAIV challenge in immunized chickens, rAPMV-3 expressing the HA protein induced higher levels HI and neutralizing antibody titers than the rNDV expressing the HA protein. These results suggest that rAPMV-3 might be a better vector for HPAIV vaccination in the field.

## Result

### Generation of rAPMV-3 expressing HA protein of H5N1 HPAIV and expression of HA protein by rAPMV-3 and rNDV

**Figure 1:**
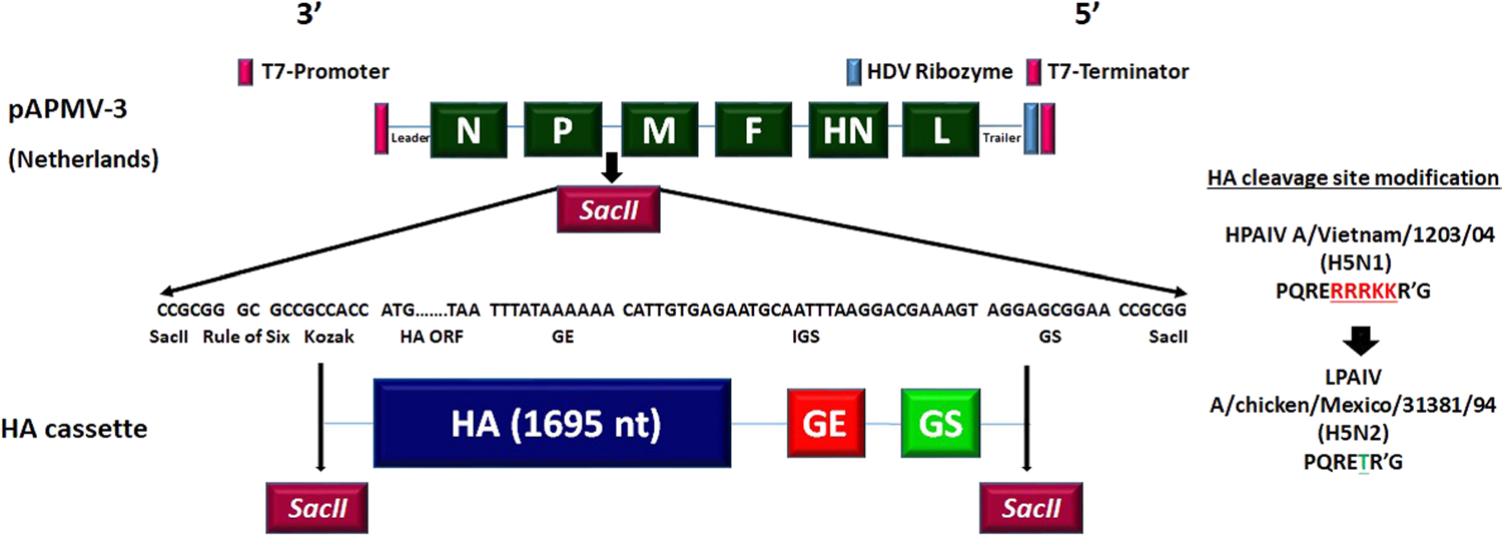
The schematic diagram for the construct of recombinant avian paramyxovirus serotype 3 (rAPMV-3) containing HA gene of HPAIV. The full-length antigenomic cDNA of APMV-3 strain Netherlands was constructed into a plasmid pBR322 previously. A transcription cassette containing ORF sequence of HA gene of H5N1 HPAIV was flanked between P and M genes of APMV-3. The HA gene transcription cassette contains sequences of following elements arranged in 3ʹ to 5ʹ order; SacII restriction enzyme site, GC nucleotides for rule of six, Kozak, HA gene ORF, GE of APMV-3 P gene that here it serves as GE for HA gene, IGS, GS of APMV-3 M gene and SacII restriction enzyme site. The polybasic cleavage site motifs (PQRERRRKKR’G) of HPAIV strain A/Vietnam/1203/2004 (H5N1) was modified to monobasic cleavage site motifs (PQRETR’G) of LPAIV strain A/Mexico/31381/94 (H5N2).

A transcription cassette containing codon optimized HA gene of H5N1 HPAIV was inserted between P and M gene of rAPMV-3 (Fig. 1). The rAPMV-3 expressing HA protein was recovered from the full length antigenomic cDNA plasmid and propagated in allantoic fluid of embryonated SPF chicken eggs. The full length antigenomic cDNA of LaSota containing HA gene plasmid that was constructed previously^32^ also was recovered previously and was used in parallel with rAPMV-3 expressing HA protein for *in vitro* and *in vivo* comparisons. The rAPMV-3 expressing HA protein and rNDV strain LaSota expressing HA protein were named rAPMV-3/HA and rNDV/HA, respectively.

**Figure 2:**
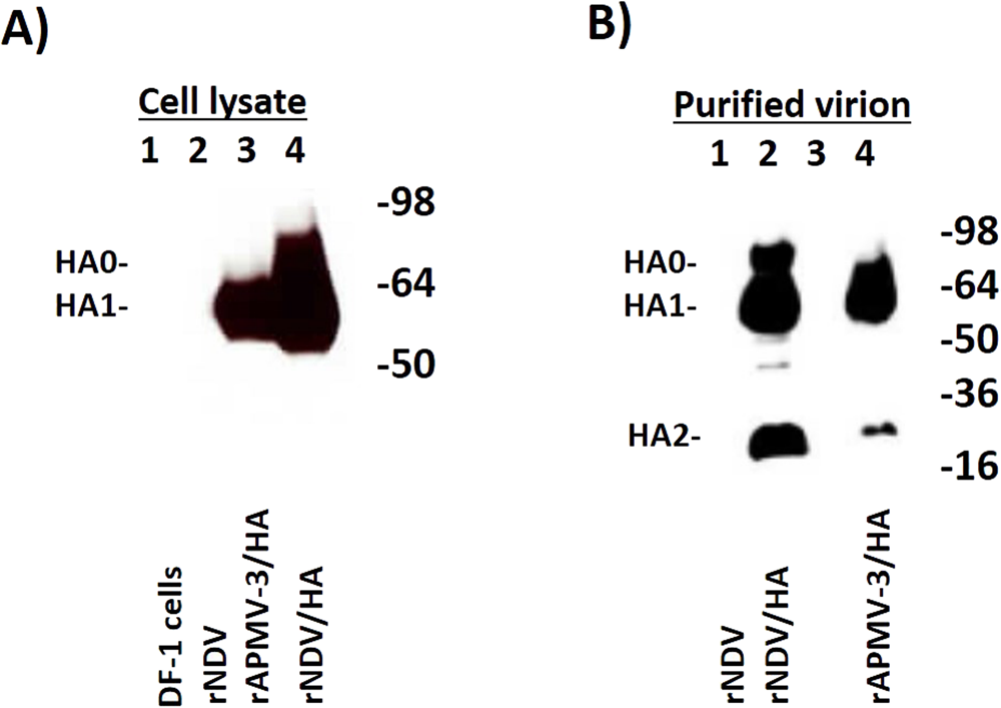
The expression of HA protein by rAPMV-3 or rNDV and incorporation of HA protein into rAPMV-3 or rNDV particles. The monolayer of DF1 cells were infected with rNDV, rNDV/HA or rAPMV-3/HA at 0.1 MOI. The cell lysates were collected 30 hours after infection. The expression of HA protein was detected by Western blot analysis using a polyclonal chicken anti H5N1 serum. (**A**) Lanes 1–4 represents; DF1 cells, rNDV, rAPMV-3 HA and rNDV/HA, respectively. The recombinant viruses were inoculated in 10-day-embryonated SPF chicken eggs, the infected allantoic fluids were collected three days post-inoculation and were partially purified. The incorporation of HA protein into rAPMV-3 or rNDV particles were detected by Western blot analysis using the mentioned serum. (**B**) The lanes 1–4 represent; rNDV, rNDV/HA, empty lane and rAPMV-3/HA, respectively. The full-length gels are presented in Supplementary Fig. S1.

The expression of HA protein by rAPMV-3/HA and rNDV/HA was detected in chicken embryo fibroblast (DF1) cell lysates by Western blot analysis (Fig. 2A). Both rAPMV-3/HA and rNDV/HA expressed HA protein efficiently. However, the expression of HA protein by rNDV/HA in DF1 cells was detected at slightly higher level than that by rAPMV-3/HA. A ~60 kDa band in Fig. 2A lane 3 represents HA1 subunit of cleaved HA0 protein expressed by rAPMV-3/HA. A ~70 kDa band and a ~60 kDa band in Fig. 2A lane 4 represent HA1 protein and uncleaved HA0 protein expressed by rNDV/HA, respectively. Lanes 1 and 2 of Fig. 2A represent DF1 cells and rNDV as controls. The incorporation of HA protein into rAPMV-3 or rNDV particles also was detected by Western blot analysis (Fig. 2B). The HA protein was incorporated into rAPMV-3/HA (Fig. 2B lane 4) and rNDV/HA (Fig. 2B lane 2) particles. Three ~70, ~60 and ~25 kDa bands represent uncleaved HA protein (HA0), HA1 subunit and HA2 subunit, respectively.

### Growth characteristics of rAPMV-3 and rNDV expressing HA protein of H5N1 HPAIV

**Figure 3:**
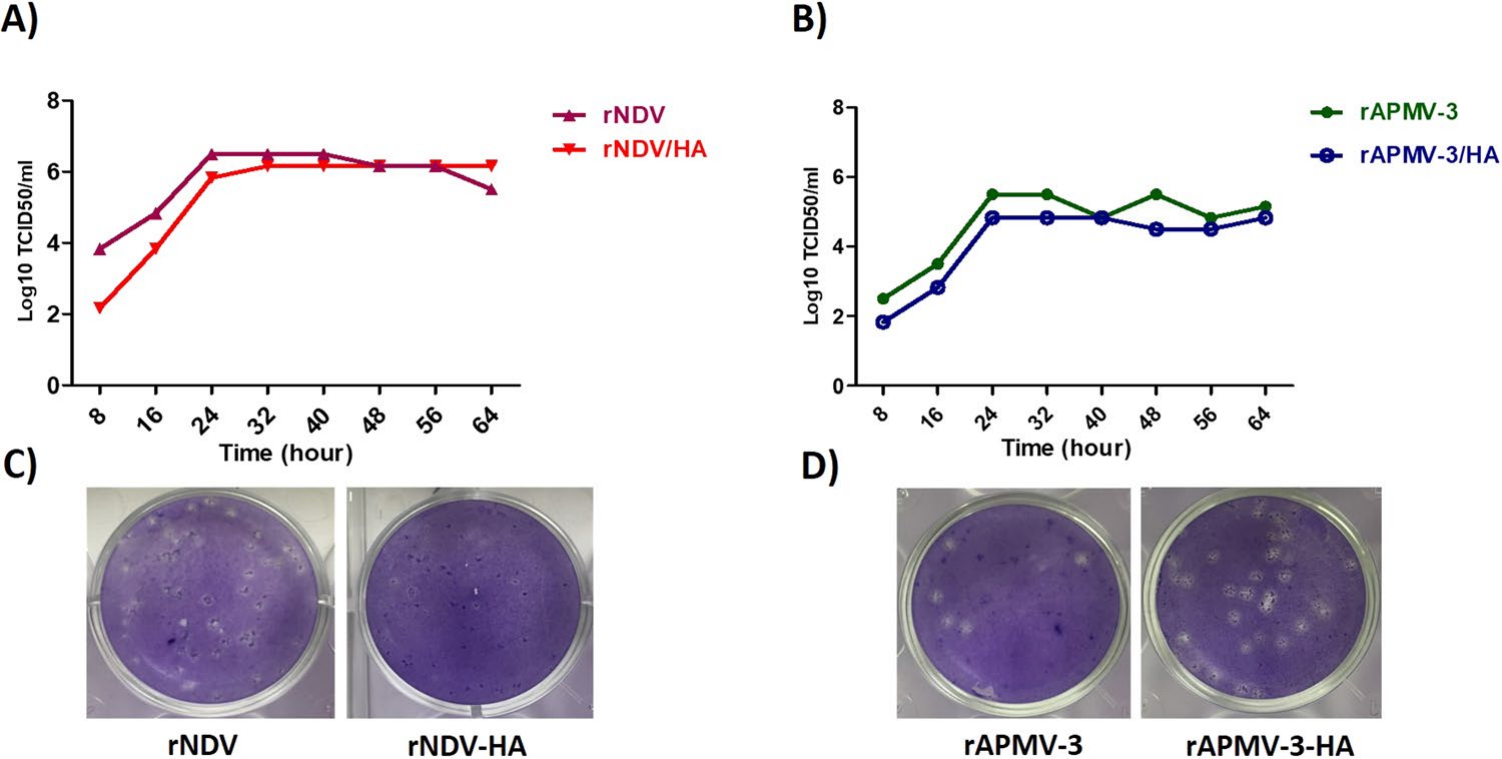
Multicycle growth kinetics and plaque morphology of rNDV or rAPMV-3 expressing HA protein in DF1 cells. The monolayers of DF1 cells were infected with rNDV, rNDV/HA, rAPMV-3 and rAPMV-3/HA at 0.1 MOI. The supernatant media containing virus were removed after one hour adsorbtion. The cells were washed and DMEM containing 10% allantoic fluid was added to cells. The titer of viruses in equal volumes of supernatants collected from infected cells in 8 hours intervals were detected by TCID_50_ using DF1 cells. (**A**,**B**) The plaque morphology of rNDV, rNDV/HA, rAPMV-3 and rAPMV-3/HA in presence of 10% allantoic fluid were evaluated in DF1 monolayers (**C**).

The rAPMV-3/HA and rNDV/HA were passaged in 10-day-old SPF embryonated chicken eggs. Both recombinant viruses replicated efficiently in eggs. However, rAPMV-3/HA replicated slightly more efficiently in eggs with titer of about 2^8^-2^9^ HAU/50 μl than rNDV/HA with titer of about 2^7^-2^8^ HAU/50 μl. Compared to rNDV/HA, rAPMV-3/HA developed larger plaques in monolayers of DF1 cells in presence of Dulbecco’s minimal essential medium (DMEM) containing 0.8% methylcellulose and 10% fresh allantoic fluid over layer (Fig. 3C). The multicycle growth kinetics of recombinant viruses showed that rNDV/HA and rAPMV-3/HA grew slightly slowly in DF1 cells than their corresponding recombinant parental viruses (Fig. 3A,B).

### The protective efficacies of rAPMV-3 and rNDV expressing HA protein against H5N1 HPAIV challenge in SPF chickens

#### HPAIV protection experiment 1

**Figure 4:**
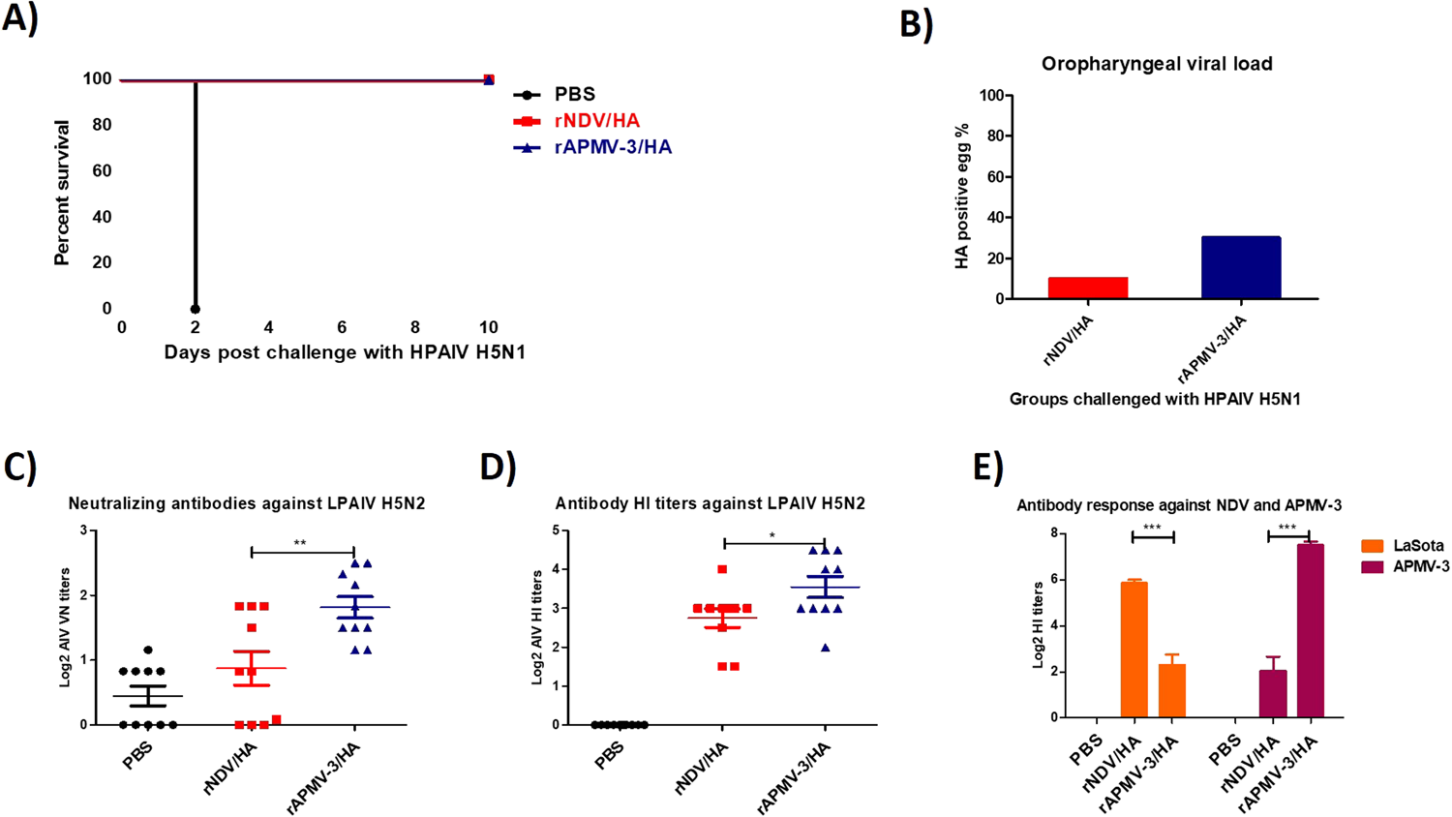
The induction of neutralizing antibodies and protective efficacy of rAPMV-3 or rNDV expressing HA protein against H5N1 HPAIV in SPF chickens (experiment 1). Day old SPF chicks were immunized with 10^6.5^ TCID_50_/bird of rNDV/HA or rAPMV-3/HA through oculonasal route. Three weeks after immunization, chickens were infected with 10^5^ EID_50_ of H5N1 HPAIV. The infected birds were observed for 10 days post-challenge and the mortality rate for each group were recorded daily. (**A**) At day four post-challenge oropharyngeal swabs were taken from survived birds and each swab sample were inoculated in one 10-day-old embryonated eggs for re-isolation of challenge virus. The infected eggs were detected by HA assay. (**B**) Three weeks post-immunization the sera were collected from all chickens and the level of antibodies induced against a H5N2 LPAIV were detected by micro-virus neutralization assay (**C**) and HI assay (**D**) and the antibody HI titers against NDV and APMV-3 also were detected by HI assay. (**E**) Serum titers are expressed as reciprocals Log2 dilution. Statistical significance among groups was: P < 0.05 (*), p < 0.01(**) and p < 0.0001 (***).

In this study four-day-old SPF chicks were immunized by the oculonasal route with rAPMV-3/HA or rNDV/HA and infected with 10^5^ 50% egg infectious dose (EID_50_)/bird of H5N1 HPAIV by the oculonasal route at three weeks after immunization. The results showed that all chickens immunized with rAPMV-3/HA or rNDV/HA were protected from mortality of HPAIV challenge until ten days post-challenge, whereas all unimmunized chickens died at day two after challenge (Fig. 4A). At day four post-challenge, the challenge virus was re-isolated from three out of ten chickens immunize with rAPMV-3/HA, while it was re-isolated from only one out of ten chickens immunized with rNDV/HA (Fig. 4B). Compared to rNDV/HA, immunization of chickens with rAPMV-3/HA induced higher neutralizing antibodies (Fig. 4C) (P < 0.05) and higher HI titers (Fig. 4D) against HPAIV and corresponding backbone vector (Fig. 4E) (P < 0.05).

#### HPAIV protection experiment 2

**Figure 5:**
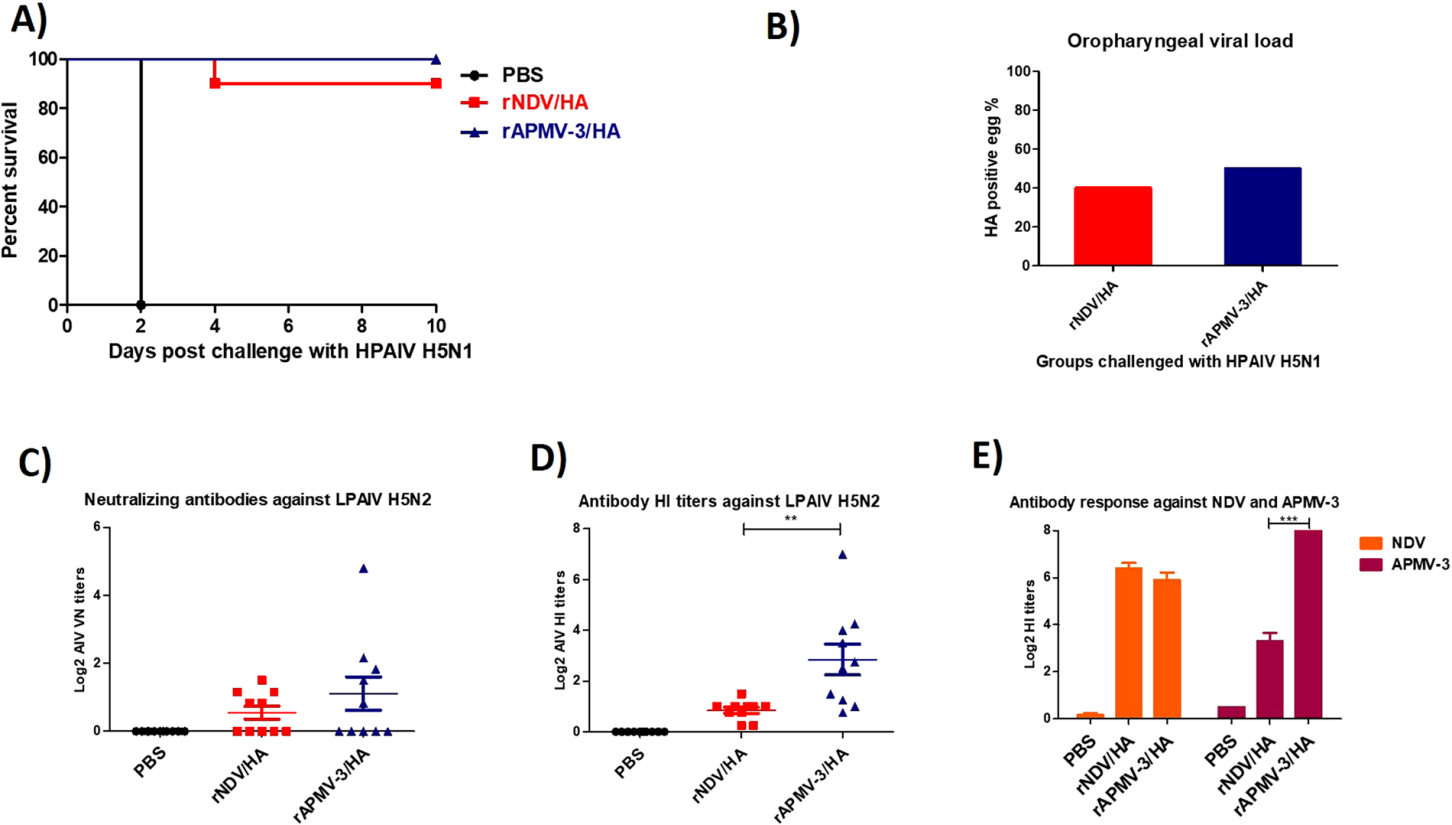
The induction of neutralizing antibodies and protective efficacy of rAPMV-3 or rNDV expressing HA protein against H5N1 HPAIV in SPF chickens (experiment 2). Day old SPF chicks were immunized with 10^6^ PFU/bird of rNDV/HA or rAPMV-3/HA through oculonasal route. Three weeks after immunization, chickens were infected with 10^6^ EID_50_ of H5N1 HPAIV. The infected birds were observed for 10 days post-challenge and the mortality rate for each group were recorded daily. (**A**) At day four post-challenge oropharyngeal swabs were taken from survived birds and each swab sample were inoculated in five 10-day-old embryonated eggs for re-isolation of challenge virus. The infected eggs were detected by HA assay. (**B**) Three weeks post-immunization the sera were collected from all chickens and the level of antibodies induced against the H5N2 LPAIV were detected by micro-virus neutralization assay (**C**) and HI assay. (**D**) The humoral antibody responses against NDV and APMV-3 also were detected by HI assay. (**E**) Serum titers are expressed as reciprocals Log2 dilution. Statistical significance among groups was: p < 0.01(**) and p < 0.0001 (***).

In this study day-old SPF chickens were immunized with rAPMV-3/HA or rNDV/HA and infected with a higher dose of 10^6^ EID_50_/bird of H5N1 HPAIV at three weeks after immunization. The results showed that all chickens immunized with rAPMV-3/HA survived from HPAIV challenge. Nine out of ten chickens immunized with rNDV/HA were protected from death of HPAIV until ten days post-challenge and one chicken died at day four after challenge. Whereas all non-vaccinated chickens died at day two after challenge. (Fig. 5A). At day four post-challenge, the challenge virus was re-isolated from five out of ten chickens immunize with rAPMV-3/HA and four out of ten chickens immunized with rNDV/HA (Fig. 5B). Compared to rNDV/HA, immunization of chickens with rAPMV-3/HA induced higher neutralizing antibodies (Fig. 5C) and higher HI titers against HPAIV (Fig. 5D) (P < 0.05) and corresponding backbone vector (Fig. 5E).

### The protective efficacies of rAPMV-3 and rNDV expressing HA protein against H5N1 HPAIV challenge in broilers

**Figure 6:**
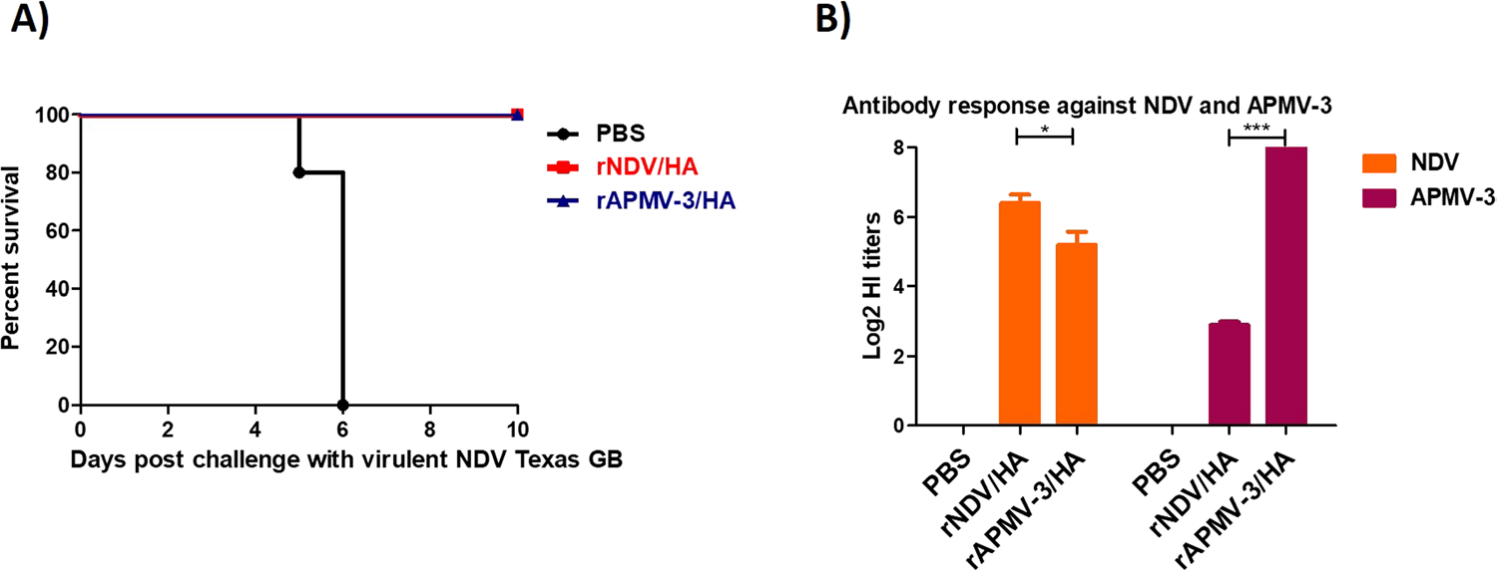
The protective efficacy of rAPMV-3 or rNDV expressing HA against H5N1 HPAIV in broilers. The groups of five broiler chickens were immunized with 10^6^ PFU/bird of rNDV/HA and/or rAPMV-3/HA based on regimens of vaccination for each group mentioned in Table 1 through oculonasal route. Chickens were infected with 10^6^ EID_50_ of H5N1 HPAIV by the oculonasal route at week five of age. The infected birds were observed for 10 days post-challenge and the mortality rate for each group were recorded daily. (**A**) Three weeks after prime-immunization and two weeks after boost-immunization the sera were collected from all chickens and the antibodies induced against a H5N2 LPAIV were detected by HI assay. (**B**) Serum titers are expressed as reciprocals Log2 dilution. Statistical significance among groups was: P < 0.05 (*).

Groups of broilers were immunized with either single or prime-boost regimens (prime immunization at day one of age and booster immunization at week three of age) of rAPMV-3/HA and/or rNDV/HA listed in Table 1 and infected with 10^6^ EID_50_/bird of H5N1 HPAIV at week five of age. The results showed that all immunized chickens with rAPMV-3/HA and/or rNDV/HA, no matter which immunization regimen, survived from HPAIV challenge. Whereas all non-vaccinated broilers died at day one after challenge (Fig. 6A). The challenge virus was re-isolated from no immunized chicken at day four after challenge.

**Table 1:** The groups of broilers immunized in single or prime-boost regimens.

Groups	Prime immunization	Boost immunization
A	PBS	PBS
B	rNDV/HA	rAPMV-3/HA
C	rNDV/HA	—
D	rAPMV-3/HA	rNDV/HA
E	rAPMV-3/HA	—
F	—	rAPMV-3/HA

**Figure 7:**
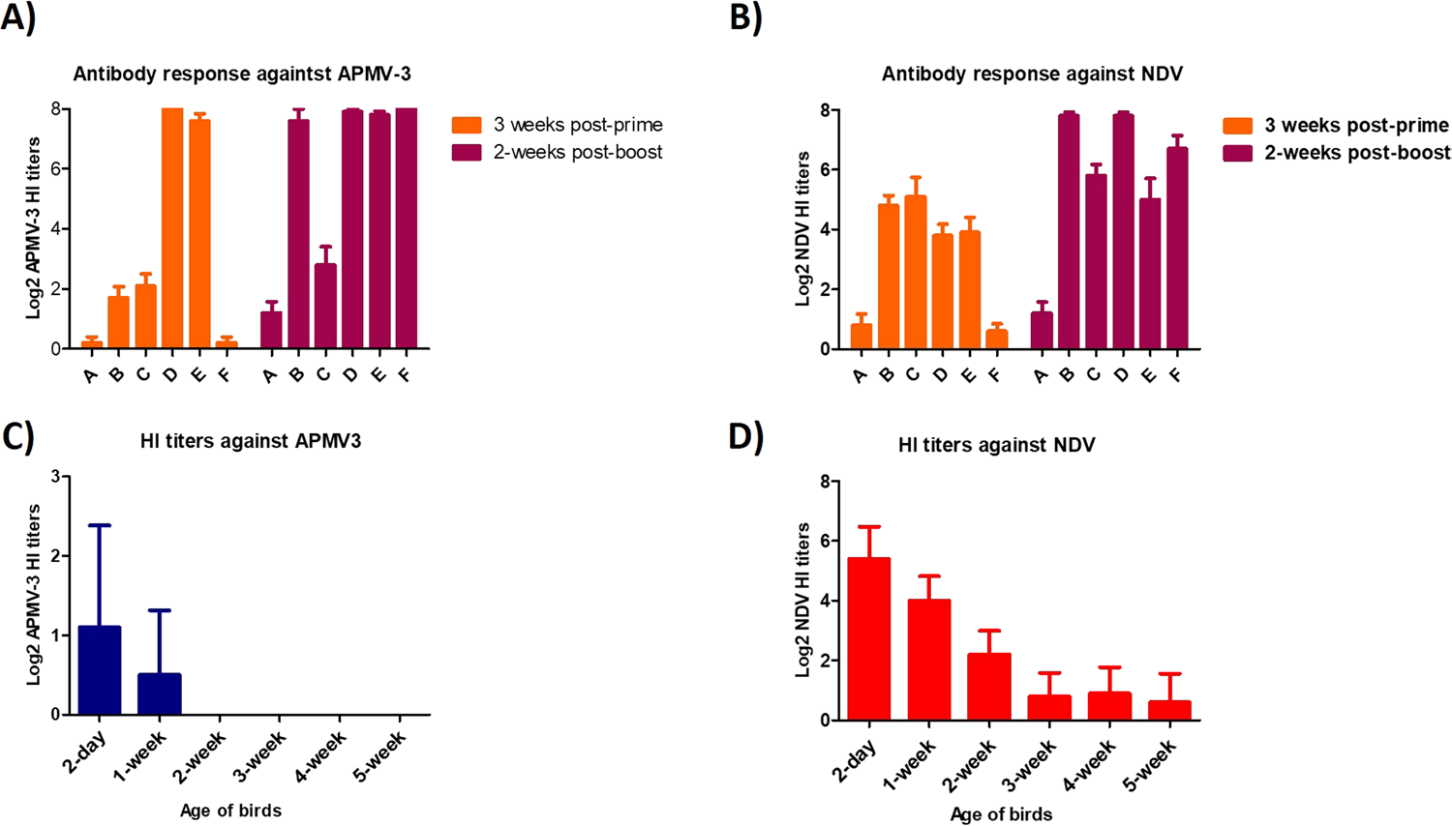
The antibody responses against APMV-3 and NDV by rAPMV-3 or rNDV expressing HA in broilers. The groups of broilers were immunized with 10^6^ TCID_50_/bird of rNDV/HA or rAPMV-3/HA based on regimen of vaccination for each group mentioned in Table 1 through oculonasal route. Three weeks after prime-immunization and two weeks after boost-immunization the sera were collected from all chickens and the antibodies induced against rAPMV-3 strain Netherlands (**A**) and rNDV strain LaSota (**B**) were detected by HI assay. The level of maternally derived antibodies (MDAs) against rAPMV-3 strain Netherlands (**C**) and rNDV strain LaSota (**D**) in non-infected broilers also were detected by HI assay. Serum titers are expressed as reciprocals Log2 dilution.

Single immunization of day old broilers with either rAPMV-3/HA or rNDV/HA induced humoral antibodies against HPAIV (Fig. 6B) and the corresponding backbone vector (Fig. 7A,B) and over time, antibody titers increased at five weeks after immunization (HI titers at five weeks post-immunization were higher than those at three weeks post-immunization) (Figs. 6B, 7A,B). Prime immunization of broilers with rAPMV-3/HA followed by a booster immunization with rNDV/HA induced higher HI titers against HPAIV compared to the prime immunization of broilers with rNDV/HA followed by a booster immunization with rAPMV-3/HA (Fig. 6B) (P < 0.05). The maternal derived antibodies (MDAs) HI titers against NDV strain LaSota was detected in non-infected control broilers at day two, weeks 1, 2, 3, 4 and 5 of age, and the titers decreased by the age (Fig. 7D). The MDAs HI titers also was detected against rAPMV-3 at day two and week one of age (Fig. 7C).

### The protective efficacies of rAPMV-3 and rNDV expressing HA protein against highly virulent NDV challenge

**Figure 8:**
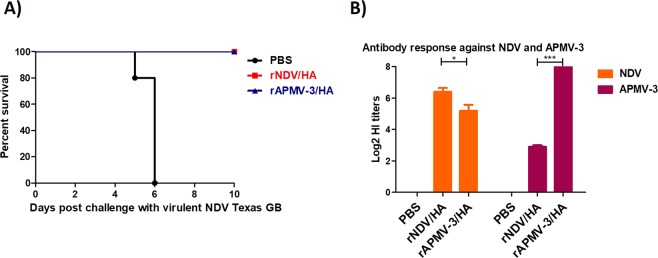
The antibody responses and the protective efficacy rAPMV-3 or rNDV expressing HA against NDV strain GB Texas in immunized SPF chickens. Four-day-old SPF chickens were immunized with 10^6.5^ TCID_50_/ml of rAPMV-3/HA or rNDV/HA through oculonasal route. Three weeks after immunization, chickens were infected with 100 CLD_50_ of NDV strain GB Texas. The infected birds were observed for 10 days post-challenge and the mortality rate clinical signs of NDV were recorded daily. (**A**) Three weeks post-immunization the sera were collected from all chickens and the level of antibodies induced against APMV-3 and NDV were detected by HI assay. (**B**) Serum titers are expressed as reciprocals Log2 dilution. Statistical significance among groups was: P < 0.05 (*) and p < 0.0001 (***).

Four-day-old SPF chicks were immunized with rAPMV-3/HA or rNDV/HA and infected with 100 50% chicken lethal dose (CLD_50_)/bird of virulent NDV at three weeks after immunization. The results showed that all chickens immunized with either rAPMV-3/HA or rNDV/HA were protected from mortality and clinical signs of virulent NDV challenge until ten days post-challenge, whereas all non-immunized chickens died at days five and six after challenge (Fig. 8A). The rAPMV-3/HA induced higher levels of HI titers against APMV-3 than the HI titers that rNDV/HA induced against NDV (Fig. 8B) (P < 0.05). Cross-reactions were observed between antibodies induced by rNDV/HA and rAPMV-3/HA against rAPMV-3 and rNDV, respectively (Fig. 8B).

## Discussion

rNDV strain LaSota has been found as a highly successful vaccine vector against HPAIV in chickens^10,17–23^. Although rNDV vector induces good local and systemic immune responses, its replication is restricted only to the respiratory tract. We therefore thought that the immune response to the HA antigen can be enhanced by using another avirulent avian paramyxovirus vector that not only replicates in the respiratory tract, but also systemically. We chose to evaluate APMV-3 as a vaccine vector, because it does not cause apparent disease in chickens, replicates well in chickens and is not a Select Agent. APMV-3 possesses a multibasic amino acid motif at the FPCS, which allows it to replicate not only in the respiratory tract, but also systemically resulting in induction of robust immune responses^28–31^. A recent study showed that rAPMV-3 vector expressing the Ebola virus glycoprotein induced high levels of mucosal and humoral neutralizing antibodies against Ebola virus in guinea pigs^33^. Both APMV-3 FPCS (A-R-P-R-G-R↓L) and virulent NDV strains FPCS (R-R-Q-K-R↓F) contain multibasic amino acids which is recognized by furin like proteases^28^. But the FPCS of virulent NDV strains contain a F residue at the first position of F1 subunit and the FPCS of avirulent NDV strains contain a L residue at this position. It has been suggested that the presence of F residue versus L residue as the first residue of F1 subunit is associated with virulence^34^. APMV-3 FPCS contains a L residue at this position. We think that although APMV-3 FPCS contains the furin recognition site (R-X-(R/K)-R↓), its lack of F residue at the first position of F1 subunit and other viral factors maybe the reason why it is avirulent in chickens. Therefore, APMV-3 can replicate systemically, but does not cause apparent disease in chickens. Hence, rAPMV-3 is an attractive vector to generate a recombinant HA-based vaccine against HPAIV. In this study, by employing reverse genetics techniques, we generated rAPMV3 strain Netherlands expressing the HA protein of H5N1 HPAIV. We then compared the efficacy of rAPMV3 expressing HA protein with that of the rNDV expressing HA protein in induction of neutralizing antibodies and protection against HPAIV in SPF and commercial chickens.

Both rAPMV-3 and rNDV expressed HA protein at high levels in DF1 cells and the HA protein was found to be incorporated into both rAPMV-3 and rNDV particles. However, the expression level of HA protein by rNDV was slightly higher than that by rAPMV-3 in DF1 cells. The higher level of HA expression by the rNDV vector maybe due to the higher level of replication of the rNDV vector *in vitro* in presence of exogenous protease. rAPMV-3 or rNDV expressing HA grew slightly slowly compared to their corresponding parental viruses in DF1 cells, which may be due to insertion of a foreign gene into the viral genome^35^. Compared to rNDV vector, rAPMV-3 vector developed large size plaques in DF1 cells and grew in embryonated chicken eggs about 1 log higher titer, as a result of the difference between FPCS of the two viruses^28,29^.

Our results showed that compared to rNDV expressing HA protein, rAPMV-3 expressing HA protein induced higher levels of neutralizing antibodies against HPAIV and against the corresponding backbone vector in SPF chickens. These results are in consistent with the result of a previous study indicating that rAPMV-3 replicates systemically than rNDV in chickens leading to induction of stronger humoral antibody response^36^. We also found that prime immunization of broilers with rAPMV-3 expressing HA protein followed by a booster immunization with rNDV expressing HA compared to immunization of broiler chickens with rNDV expressing HA protein followed by a booster immunization with rAPMV-3 expressing HA protein resulted in higher levels of humoral antibody induced against HPAIV. These results indicate that day-old chicks contain maternally derived NDV antibodies which can inhibit the replication of rNDV expressing HA but is unable to affect the replication of rAPMV-3/HA. At 3-week of age the maternally derived NDV antibodies are waned, which no more affect the replication of rNDV expressing HA. Hence, a stronger immune response to HA is achieved when rAPMV-3/HA is used for prime immunization followed by boosting immunization at 3-week of age with rNDV expressing HA. Overcome of pre-existing antibodies has also been reported for other paramyxovirus vectors^37–39^. Furthermore, these results indicate that the rAPMV-3/HA has high efficiency replication in a greater range of chick organs. Therefore, at 5 weeks age, the higher levels of antibodies were detected in chickens that were prime-immunized with rAPMV-/HA then boosted with rNDV/HA than chickens that prime immunized with rNDV/HA then boosted with rAPMV-3/HA. Because rAPMV-3/HA induced antibodies for a longer period (5 weeks) in chickens that prime immunized with rAPMV-3 than the period (2 weeks) in chickens that boost immunized with rAPMV-3/HA.

Immunization of chickens with rAPMV-3 expressing HA protein or rNDV expressing HA protein provided complete protection from death following HPAIV challenge. These results suggest that the immunity provided by rAPMV-3 or rNDV expressing HA protein was enough to provide protection against HPAIV challenge in chickens, in consistent with previous reports^3,17–23^. Immunization of SPF chickens with either rAPMV-3 expressing HA or rNDV expressing HA decreased the post-HPAIV challenge oropharyngeal viral load, significantly. Despite the higher levels of neutralizing antibodies induced against HPAIV by rAPMV-3 expressing HA protein and by rNDV expressing HA protein, neither rAPMV-3 nor rNDV vector provided sterilizing protection against HPAIV in SPF chickens in two experimental challenges. In the case of broilers, single immunization or prime-boost immunization of chickens with rAPMV-3 or rNDV expressing HA protein resulted in eliciting sterilizing immunity. The absent of viral shedding following HPAIV challenge in broilers could be due to a low dose of challenge, the higher age of chickens at time of challenge, and/or the low number of eggs per swab sample for re-isolation of challenge virus. These results support the generally accepted view that it is difficult to achieve sterilizing protection against HPAIV by vaccination^7^. However, in a very well optimized challenge experiment a very low level of challenge virus may be isolated from chickens immunized with a highly efficacious vaccine that may be considered as sterilizing protection.

Both rAPMV-3 and rNDV vectors expressing the HA protein did not completely stopped challenge virus shedding. We do not consider this to be a major deficiency of these vector vaccines, because H5N1 HPAIV is a fast replicating and a highly infectious virus; therefore, complete cessation of replication of 10^6^ EID_50_ of H5N1 HPAIV delivered directly into the nasal tract of a chicken will require very high levels of local and systemic immunity. In field conditions, vaccinated chickens will not encounter such high dose of infectious HPAIV. Therefore, we think that the levels of neutralizing antibody titers elicited by both vectors will be sufficient to provide complete protection against HPAIV in the field. Surprisingly, a slightly higher challenge virus shedding was found in chickens vaccinated with rAPMV-3 expressing HA than with rNDV expressing HA. This could have occurred due to some unknown reason.

There are several reasons why we think rAPMV-3/HA is a better vaccine than rNDV/HA in field conditions in poultry. Although, immunization of chickens with rAPMV-3/HA and rNDV/HA provided comparable protection against HPAIV, rAPMV-3/HA replicated more efficiently than rNDV/HA in chickens leading to the induction of significant higher levels of neutralizing antibodies. In the field mass-vaccination conditions, birds are vaccinated via spray method instead of direct intranasal and intraocular routes, chickens uptake lower titers of vaccine. Therefore, low dose of rAPMV-3/HA due to more efficient replication in chickens can induce higher levels of neutralizing antibodies and might provide better protection. However, the exact evaluation of comparative protective efficacies of rAPMV-3/HA and rNDV/HA under field conditions, will require large scale studies. Another advantage of rAPMV-3/HA over rNDV/HA is that it replicates to higher titer in embryonated chicken eggs, which is a merit for mass-production of vaccine. Furthermore, rAPMV-3/HA is not inhibited by NDV maternally derived antibodies; therefore, can be an efficient vaccine for day-old broiler chicks.

Our NDV challenge results showed that immunization of SPF chickens with rAPMV-3 or rNDV expressing HA protein provided complete protection from death against highly virulent NDV strain GB Texas challenge. Interestingly, there are only 31% and 34% amino acid identities between F and HN proteins of APMV-3 and NDV, respectively, but they provide cross-protection^28^. This protection was likely provided by some cross-reactive neutralizing epitopes in surface glycoproteins of APMV-3, which has been reported previously^40^.

In summary, the results of this study showed that immunization of chickens with rAPMV-3 expressing HA protein or rNDV expressing HA protein provided complete protection from disease following HPAIV challenge. But the results showed that immunization of chickens with rAPMV-3 expressing HA protein induced higher levels of neutralizing antibodies than the levels induced by rNDV expressing HA protein. Taken together these results suggest that a rAPMV-3 expressing HA protein might be a better vaccine for mass-vaccination of commercial chickens in field conditions.

## Materials and Methods

### Cells and viruses

Human epidermoid carcinoma (HEp-2) cells and DF1 cells were obtained from the American Type Culture Collection (ATCC, Manassas, VA) to recover recombinant viruses by reverse genetics and for *in vitro* characterization of recovered viruses, respectively. The Madin-Darby canine kidney (MDCK) cells was also obtained from ATCC to perform virus-neutralization test against LPAIV strain A/Mallard/Pennsylvania/10218/1984 (H5N2). HEp-2 and DF1 cells were grown in Dulbecco’s minimal essential medium (DMEM) containing 10% fetal bovine serum (FBS); and MDCK cells were grown in DMEM containing 5% FBS. Recombinant viruses were propagated in allantoic fluids of 10-day-old embryonated SPF chicken eggs (Charles Rivers, MA). HPAIV strain A/Vietnam/1203/2004 (H5N1) was propagated in 10-day-old embryonated SPF chicken eggs, aliquoted and stored at −70 °C in Biosafety Level-3 plus facilities. The stored virus stock was used as challenge virus and its end point titer was determined by EID_50_. The H5N2 LPAIV was propagated in 10-day-old embryonated SPF chicken eggs, aliquoted and stored at −70 °C in Biosafety level-2 facility. The stored virus stock was used in micro-neutralization assay and its end point titer was determined by 50% tissue culture infectious dese (TCID_50_) in MDCK.

### Construction of rAPMV-3 expressing HA protein of H5N1 HPAIV

A plasmid containing full-length antigenomic cDNA of APMV-3 strain Netherlands was constructed previously^41,42^ and later the viral protein 2 (VP2) gene of infectious bursal disease virus (IBDV) was flanked between P and M genes of APMV-3 using this plasmid. Here we replaced the VP2 gene with HA gene of HPAIV strain A/Vietnam/1203/2004 (H5N1) using *SacII* restriction enzyme site. The HA gene was codon optimized for higher level of expression in human (mammalian) cells. A transcription cassette containing ORF sequence of HA gene of H5N1 HPAIV was amplified from a plasmid of full length antigenomic cDNA of NDV containing codon optimized HA gene of HPAIV strain A/Vietnam/1203/2004 (H5N1). The polybasic cleavage site motifs (PQRERRRKKR’G) of HPAIV strain A/Vietnam/1203/2004 (H5N1) was modified to monobasic cleavage site motifs (PQRETR’G) of LPAIV strain A/Mexico/31381/94 (H5N2). The HA gene transcription cassette contained nucleotide sequences of following genetic elements arranged in 3ʹ to 5ʹ order; *SacII* restriction enzyme site, GC nucleotides for rule of six, Kozak, HA gene open reading frame (ORF), gene end (GE) of P gene of APMV-3 (here it serves as GE for HA gene), 31 nucleotides of APMV-3 intergenic sequences (IGS), gene start (GS) of APMV-3 M gene and *SacII* restriction enzyme site. Following the cloning of the amplified fragment of HA gene transcription cassette into pGEM®–T Easy vector (Promega Corporation), it was digested by *SacII* restriction enzyme, and re-cloned between P and M genes of APMV-3 instead of VP2 gene which was inserted between P and M genes previously. The recombinant APMV-3 expressing HA protein was recovered by co-transfection of the plasmid of full-length anitigenomic cDNA of APMV-3 containing HA gene and supporting plasmids containing nuclocapsid protein (N), phosphoprotein (P) or large polymerase protein (L) of APMV-3 strain Netherlands into HEp-2 cells using reverse genetic techniques as described previously^43^. The rAPMV-3 expressing HA protein and rNDV strain LaSota expressing HA protein were named rAPMV-3/HA and rLaSota/HA, respectively.

### Expression of HA protein by rAPMV-3 and rNDV expressing HA protein of H5N1 HPAIV

The monolayer of DF1 cells were infected with rNDV, rNDV/HA and rAPMV-3/HA in presence of 10% allantoic fluid. The DF1 cell lysates were collected 30 hours after infection. A polyclonal chicken anti H5N1 serum was used to detect expression of HA protein. In order to detect incorporation of HA protein into rAPMV-3 or rNDV particles, the recombinant viruses were inoculated in 10-day-old embryonated SPF chicken eggs. Three days after inoculation the allantoic fluids were harvested and centrifuged at 1500 rpm for 10 minutes to remove the cell debris. The allantoic fluids containing recombinant particles were ultra-centrifuged at 28000 rpm (rotor SW28) for 2:30 hours at 4 °C through 30% sucrose cushion. The sedimented pellets containing partially purified virus particles were dissolved in 50 μl phosphate buffer saline (PBS). The Western blot analysis using above serum was utilized to detect the incorporation of HA protein into rAPMV-3 or rNDV particles.

### Growth characteristics of rAPMV-3 and rNDV expressing HA protein of H5N1 HPAIV

The recombinant viruses were passaged in 10-day-old embryonated SPF chicken eggs. The viruses with high HA titers were harvested and stored at −70 °C in aliquots. The titer of each stored virus was determined by plaque assay in presence of 10% allantoic fluid. Then the monolayers of DF1 cells in a 6-well tissue culture plate were infected with rNDV, rNDV/HA, rAPMV-3 and rAPMV-3/HA at 0.1 MOI. The over layered medium was removed after one-hour adsorption and replaced with fresh DMEM containing 10% allantoic fluid following washing the monolayers with DMEM. Volumes of 200 μl were collected from each well and replaced with fresh medium containing 10% allantoic fluid in 8 hours intervals. The collected supernatants were stored at −70 °C and the titer of viruses in each sample was detected by TCID_50_ using DF1 cells. The titer of each sample was calculated using a formula derived from Reed and Muench and Spearman-Karber methods^44^.

### The protective efficacies of rAPMV-3 and rNDV expressing HA protein against H5N1 HPAIV challenge in SPF chickens

#### H5N1 HPAIV protection experiment 1

Total number of 30 four-day-old white leghorn SPF chicks, purchased from Charles Rivers, were divided in three groups, each group ten chicks. The chicks of first and second groups were immunized with 10^6.5^ TCID_50_/bird, 160 μl in volume, of rNDV expressing HA protein and rAPMV-3 expressing HA protein, respectively. The birds of the third group were inoculated with 160 μl PBS through oculonasal route. Three weeks after immunization, birds were transferred to the Biosafety level-3 plus facility and were infected with 10^5^ EID_50_ of HPAIV strain A/Vietnam/1203/2004 (H5N1) by the oculonasal route. The infected chickens were observed daily for death and clinical signs of H5N1 HPAIV. At day four post-challenge, oropharyngeal swabs were taken from chickens and were kept in three ml cold DMEM containing 10 X antibiotics and amphotericin B. Each swab sample was centrifuged at 1500 rpm for 10 minutes and 200 μl of supernatant of each swab sample was inoculated into one 10-day-old embryonated SPF chicken egg. Three days post-inoculation the allantoic fluids were tested by HA assay for the presence of challenge virus.

#### H5N1 HPAIV protection experiment 2

Total number of 30 two-day-old white leghorn SPF chicks, purchased from Charles Rivers, were divided in three groups, each group ten chicks. The chicks of the first and the second groups were immunized with 10^6^ PFU/bird, 200 μl in volume, of rNDV expressing HA protein and rAPMV-3 expressing HA protein, respectively. The birds of the third group were inoculated with 200 μl PBS through oculonasal route. Three weeks after immunization, 29 birds (one bird from PBS group died during the immunization time) were transferred to the Biosafety level-3 facilities and were infected with 10^6^ EID_50_ of HPAIV strain A/Vietnam/1203/2004 (H5N1) by the oculonasal route. The infected chickens were observed daily for death and clinical signs of H5N1 HPAIV. At day four post-challenge, oropharyngeal swabs were taken from chickens and were kept into three ml cold DMEM containing 10X antibiotics and amphotericin B. Each swab sample was centrifuged at 1500 rpm for 10 minutes and five 10-day-old embryonated SPF chicken eggs were inoculated with supernatant of each swab sample, 200 μl per egg. Three days post-inoculation the allantoic fluids were tested by HA assay for the presence of challenge virus.

### The protective efficacies of rAPMV-3 and rNDV expressing HA protein against H5N1 HPAIV challenge in broilers

Total number of 30 two-day-old broilers were divided into six groups, five each. In a single or prime-boost immunization regimen (prime at day one and boost at week three of age), chicks were immunized with 10^6^ TCID_50_/bird, 200 μl in volume, of rNDV expressing HA protein or rAPMV-3 expressing HA protein by the oculonasal route based on the immunization regimen for each group listed in Table 1. At week five of age (five weeks-post prime immunization and two weeks post-boosting), birds were transferred to the Biosafety level-3 plus facilities and were infected with 10^6^ EID_50_ of HPAIV strain A/Vietnam/1203/2004 (H5N1) by the oculonasal route. The infected chickens were observed daily for death and clinical signs of H5N1 HPAIV. At day four post-challenge, oropharyngeal swabs were taken from chickens and were kept into three ml cold DMEM containing 10X antibiotics and amphotericin B. Each swab sample was centrifuged at 1500 rpm for 10 minutes and 200 μl of supernatant of each swab sample were inoculated in one 10-day-old embryonated SPF chicken egg. Three days post-inoculation the allantoic fluids were tested by HA assay for the presence of challenge virus.

### The protective efficacies of rAPMV-3 and rNDV expressing HA protein against virulent NDV challenge in SPF chickens

Total number of 15 four-day-old white leghorn SPF chicks, purchased from Charles Rivers, were divided into three groups, five each. The chicks of the first and the second groups were immunized with 10^6.5^ TCID_50_/bird, 160 μl in volume, of rNDV expressing HA protein and rAPMV-3 expressing HA protein, respectively. The birds of the third group were inoculated with 200 μl PBS through oculonasal route. Three weeks after immunization, birds were transferred to the Biosafety level-3 facility and were infected with 100 CLD_50_ of virulent NDV strain GB Texas by the oculonasal route. The infected chickens were observed daily for death and clinical signs of NDV.

### Serological analysis

The antibodies induced against the HA protein of H5N1 virus in sera collected from immunized chickens were assessed by hemagglutination inhibition (HI) assay using an antigenically related H5N2 LPAIV^3^. The LPAIV strain A/Mallard/Pennsylvania/10218/1984 (H5N2) was used instead of H5N1 HPAIV, because the experiment can be conducted at biosafety level-2 facility. The A/Mallard/Pennsylvania/10218/1984 (H5N2) virus is antigenically similar to A/Vietnam/1203/2004 (H5N1) virus (90% identity between HA protein amino acids). The neutralizing antibodies induced against the LPAIV H5N2 in sera were assessed by micro-virus neutralization assay using a standard protocol. Briefly, sera were incubated at 56 °C for 30 minutes. Serial two-fold dilution of each sera were prepared. 25 μl of each dilution and 25 μl of H5N2 LPAIV stock containing 10^2^ TCID_50_ of virus were mixed and incubated at 37 °C for 90 minutes. The titer of H5N2 LPAIV was determined by TCID_50_ on MDCK cells in presence of DMEM containing 1 μg/ml N-tosyl-l-phenylalanine chloromethyl ketone (TPCK)-treated trypsin. The supernatant of monolayer of MDCK cell in 96-well plates were removed and cells were washed with DMEM. First 50 μl of DMEM were added to each well. Then 50 μl mixture of serum and virus was added to each well, three wells per each dilution of serum, and incubated at 37 °C. After one-hour adsorption, the supernatant of wells was removed, and cells were washed with DMEM. One hundred μl DMEM containing 1 μg/ml TPCK-treated trypsin was added to each well and incubated at 37 °C. Three days after incubation, 50 μl of supernatant of each well were assessed for presence of LPAIV strain H5N2 virus by HA assay using 1% chicken RBC. The titer of neutralizing antibodies in each serum were calculated using a formula derived from Reed and Muench and Spearman-Karber methods^44^. The antibodies induced against NDV strain LaSota and rAPMV-3 strain Netherlands in sera of immunized chickens also were assessed by HI assay using the protocol of OIE (8 HAU antigen was used in HPAIV protective experiment-1 HI assay and 4 HAU antigen was used in other HI assays)^45^.

All animal experiments involving rAPMV-3 and avirulent NDV strain LaSota were performed in our USDA approved Biosafety level-2 and Biosafety level-2 plus facilities and all animal experiments involving HPAIV and virulent NDV were conducted in our USDA approved Biosafety level-3 plus facilities following the guidelines and approval of the Institutional of Animal Care and Use Committee (IACUC), University of Maryland. All experiments were approved by IACUC and conducted following the guidelines.

### Statistical analysis

Data were analyzed among groups by One-Way-ANOVA test. The t-test was used to compare two groups. To avoid bias, HPAIV and NDV challenge experiments were designed as blinded studies.

## Supplementary information

Supplementary Data.

## References

[1] International Committee on Taxonomy of Viruses (ICTV), https://talk.ictvonline.org/taxonomy (2018).

[2] Palese, P. & Shaw, M. L. Orthomyxoviridae: The Viruses and Their Replication. In *Fields Virology* (ed. Knipe, D. M. & Howley, P. M.) 1151–1186. (Lippincott Williams & Wilkins, 2013).

[3] OIE (World Organization for Animal Health). Avian Influenza In OIE Terrestrial Manual, Chapter2.3.4, http://www.oie.int/fileadmin/Home/eng/Health_standards/tahm/2.03.04_AI.pdf (2015).

[4] Suarez, D. L. Influenza A Virus. In *Animal Influenza* (ed. Swayne, D. E.) 3–30. (Wiley- Blackwell, 2013).

[5] Sutton TC (2018). The Pandemic Threat of Emerging H5 and H7 Avian Influenza Viruses. Viruses..

[6] Harfoot R, Webby RJ (2017). H5 influenza, a global update. J. Microbiology..

[7] Swayne DE (2012). Impact of vaccines and vaccination on global control of avian influenza. Avian Disease..

[8] Swayne DE, Pavade G, Hamilton K, Vallat B, Miyagishima K (2011). Assessment of national strategies for control of high-pathogenicity avian influenza and low-pathogenicity notifiable avian influenza in poultry, with emphasis on vaccines and vaccination. Rev. Sci. Tech..

[9] Swayne DE (2006). Principles for Vaccine Protection in Chickens and Domestic Waterfowl against Avian Influenza Emphasis on Asian H5N1 High Pathogenicity Avian Influenza. Ann. N.Y. Acad. Sci..

[10] Veits J (2006). Newcastle disease virus expressing H5 hemagglutinin gene protects chickens against Newcastle disease and avian influenza. Proc. Natl. Acad. Sci..

[11] Balzli CL (2018). The efficacy of recombinant turkey herpesvirus vaccines targeting the H5 of highly pathogenic avian influenza virus from the 2014–2015 North American outbreak. Vaccine..

[12] Bublot M (2007). Efficacy of a fowl pox-vectored avian influenza H5 vaccine against Asian H5N1 highly pathogenic avian influenza virus challenge. Avian Dis..

[13] Toro H, Tang DC (2009). Protection of chickens against avian influenza with non-replicating adenovirus-vectored vaccine. Poul. Sci..

[14] Pavlova SP, Veits J, Mettenleiter TC, Fuchs W (2009). Live vaccination with an H5-hemagglutinin-expressing infectious laryngotracheitis virus recombinant protects chickens against different highly pathogenic avian influenza viruses of the H5 subtype. Vaccine..

[15] Cui H (2013). Avirulent Marek’s disease virus type 1 strain 814 vectored vaccine expressing avian influenza (AI) virus H5 haemagglutinin induced better protection than turkey herpesvirus vectored AI vaccine. PLoS One..

[16] Li Z (2013). Efficacy of Parainfluenza virus 5 mutants expressing hemagglutinin from H5N1 influenza A virus in mice. J. Virology..

[17] Suarez DL, Pantin-Jackwood MP (2017). Recombinant viral-vectored vaccines for the control of avian influenza in poultry. Veterinary Microbiology..

[18] Römer-Oberdörfer A, Veits J, Helferich D, Mettenleiter TC (2008). Level of protection of chickens against highly pathogenic H5 avian influenza virus with Newcastle disease virus based live attenuated vector vaccine depends on homology of H5 sequence between vaccine and challenge virus. Vaccine..

[19] Nayak B (2010). Immunization of chickens with Newcastle disease virus expressing H5 hemagglutinin protects against highly pathogenic H5N1 avian influenza viruses. PLoS One..

[20] Nayak B (2010). Contributions of the avian influenza virus HA, NA, and M2 surface proteins to the induction of neutralizing antibodies and protective immunity. J. Virol..

[21] Lardinois A, Steensels M, Lambrecht B, Desloges N (2012). Potency of a recombinant NDV-H5 vaccine against various HPAI H5N1 virus challenges in SPF chickens. Avian Dis..

[22] Cornelissen LAHM (2012). Protective efficacy of Newcastle disease virus expressing soluble trimeric hemagglutinin against highly pathogenic H5N1 influenza in chickens and mice. PLoS One..

[23] Ma J (2017). Newcastle disease virus-based H5 influenza vaccine protects chickens from lethal challenge with a highly pathogenic H5N2 avian influenza virus. NPJ Vaccines..

[24] Toyoda T, Sakaguchi T, Imai K (1987). Structural comparison of the cleavage-activation site of the fusion glycoprotein between virulent and avirulent strains of Newcastle disease virus. Virology..

[25] Nagai Y (1995). Virus activation by host proteinases. A pivotal role in the spread of infection, tissue tropism and pathogenicity. Microb. Immunol..

[26] Nagai Y, Klenk HD, Rott R (1976). Proteolytic cleavage of the viral glycoproteins and its significance for the virulence of Newcastle disease virus. Virology..

[27] Bukreyev A (2005). Recombinant Newcastle disease virus expressing a foreign viral antigen is attenuated and highly immunogenic in primates. J. Virol..

[28] Samal, S. K. Newcastle disease and related avian paramyxoviruses. In *the biology of paramyxoviruses*. (ed. Smal, S. K.) 69–114. (Caister Academic Press, 2011).

[29] Alexander DJ (1980). Avian paramyxoviruses. Veterinary Bull..

[30] Kim SH, Xiao S, Shive H, Collins PL, Samal SK (2012). Replication, neurotropism, and pathogenicity of avian paramyxovirus serotypes 1-9 in chickens and ducks. PLoS one..

[31] Kumar S, Militino DF, Nayak B, Collins PL, Samal SK (2010). Experimental avian paramyxovirus serotype-3 infection in chickens and turkeys. Veterinary Research..

[32] Kim SH, Paldurai A, Xiao S, Collins PL, Samal SK (2014). Modified Newcastle disease virus vectors expressing the H5 hemagglutinin induce enhanced protection against highly pathogenic H5N1 avian influenza virus in chickens. Vaccine..

[33] Yoshida A (2019). Novel avian paramyxovirus-based vaccine vectors expressing the Ebola virus glycoprotein elicit mucosal and humoral immune responses in guinea pigs. Sci. Reports..

[34] Kattenbelt JA, Stevens MP, Gould AR (2006). Sequence variation in the Newcastle disease virus genome. Virus Res..

[35] Bukreyev A, Skiadopoulos MH, Murphy BR, Collins PL (2006). Nonsegmented negative-strand viruses as vaccine vectors. J. Virol..

[36] Nayak B (2012). Avian paramyxovirus serotypes 2-9 (APMV-2-9) vary in the ability to induce protective immunity in chickens against challenge with virulent Newcastle disease virus (APMV-1). Vaccine..

[37] Kim SH, Samal SK (2019). Innovation in Newcastle Disease Virus Vectored Avian Influenza Vaccines. Viruses..

[38] Kim SH, Samal SK (2017). Heterologous prime-boost immunization of Newcastle disease virus vectored vaccines protected broiler chickens against highly pathogenic avian influenza and Newcastle disease viruses. Vaccine.

[39] Chen Zhenhai, Xu Pei, Salyards Gregory W., Harvey Stephen B., Rada Balazs, Fu Zhen F., He Biao (2012). Evaluating a Parainfluenza Virus 5-Based Vaccine in a Host with Pre-Existing Immunity against Parainfluenza Virus 5. PLoS ONE.

[40] Alexander DJ, Chettle NJ, Parsons G (1979). Resistance of chickens to challenge with the virulent Herts 33 strain of Newcastle disease virus induced by prior infection with serologically distinct avian paramyxoviruses. Res. Vet. Sci..

[41] Yoshida A, Samal SK (2017). Avian Paramyxovirus Type-3 as a Vaccine Vector: Identification of a Genome Location for High Level Expression of a Foreign Gene. Front. microbiology..

[42] Kumar S, Nayak B, Collins PL, Samal SK (2011). Evaluation of the Newcastle disease virus F and HN proteins in protective immunity by using a recombinant avian paramyxovirus type 3 vector in chickens. J. Virol..

[43] Huang Z, Krishnamurthy S, Panda A, Samal SK (2001). High-level expression of a foreign gene from the most 39-proximal locus of a recombinant Newcastle disease virus. J. Gen. Virol..

[44] Ramakrishnan MA (2016). Determination of 50% endpoint titer using a simple formula. World J. Virol..

[45] OIE (World Organization for Animal Health). Newcastle disease, In OIE Terrestrial Manual, Chapter2.3.14, http://www.oie.int/fileadmin/Home/eng/Health_standards/tahm/2.03.14_NEWCASTLE_DIS.pdf (2012).

