# Author Correction: A recombinant avian paramyxovirus serotype 3 expressing the hemagglutinin protein protects chickens against H5N1 highly pathogenic avian influenza virus challenge

**DOI:** 10.1038/s41598-020-61547-5

**Published:** 2020-03-06

**Authors:** Edris Shirvani, Berin P. Varghese, Anandan Paldurai, Siba K. Samal

**Affiliations:** Virginia-Maryland College of Veterinary Medicine, University of Maryland, College Park, MD USA

Correction to: *Scientific Reports* 10.1038/s41598-020-59124-x, published online 10 February 2020

In this Article, Figure 6 is a duplication of Figure 8. The correct Figure 6 appears below as Figure 1.

**Figure 1:**
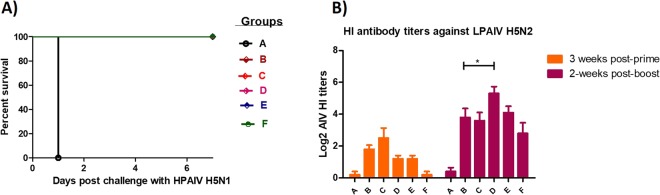
.

