# Supplementary material for: A recombinant avian paramyxovirus serotype 3 expressing the hemagglutinin protein protects chickens against H5N1 highly pathogenic avian influenza virus challenge
Source: Sci Rep. 2020 Feb 10;10:2221. doi: 10.1038/s41598-020-59124-x (PMC7010735; doi:10.1038/s41598-020-59124-x)
Supplement: Supplementary file 1 — Supplementary Data. [file 41598_2020_59124_MOESM1_ESM.docx]

**Supplementary Data**

**A recombinant avian paramyxovirus serotype 3 expressing the hemagglutinin protein protects chickens against H5N1 highly pathogenic avian influenza virus challenge.**

**Edris Shirvani, Berin P. Varghese, Anandan Paldurai, and Siba K. Samal ***

**Virginia-Maryland College of Veterinary Medicine, University of Maryland, College Park, MD, USA**

***Corresponding author,** [ssamal@umd.edu](mailto:ssamal@umd.edu)


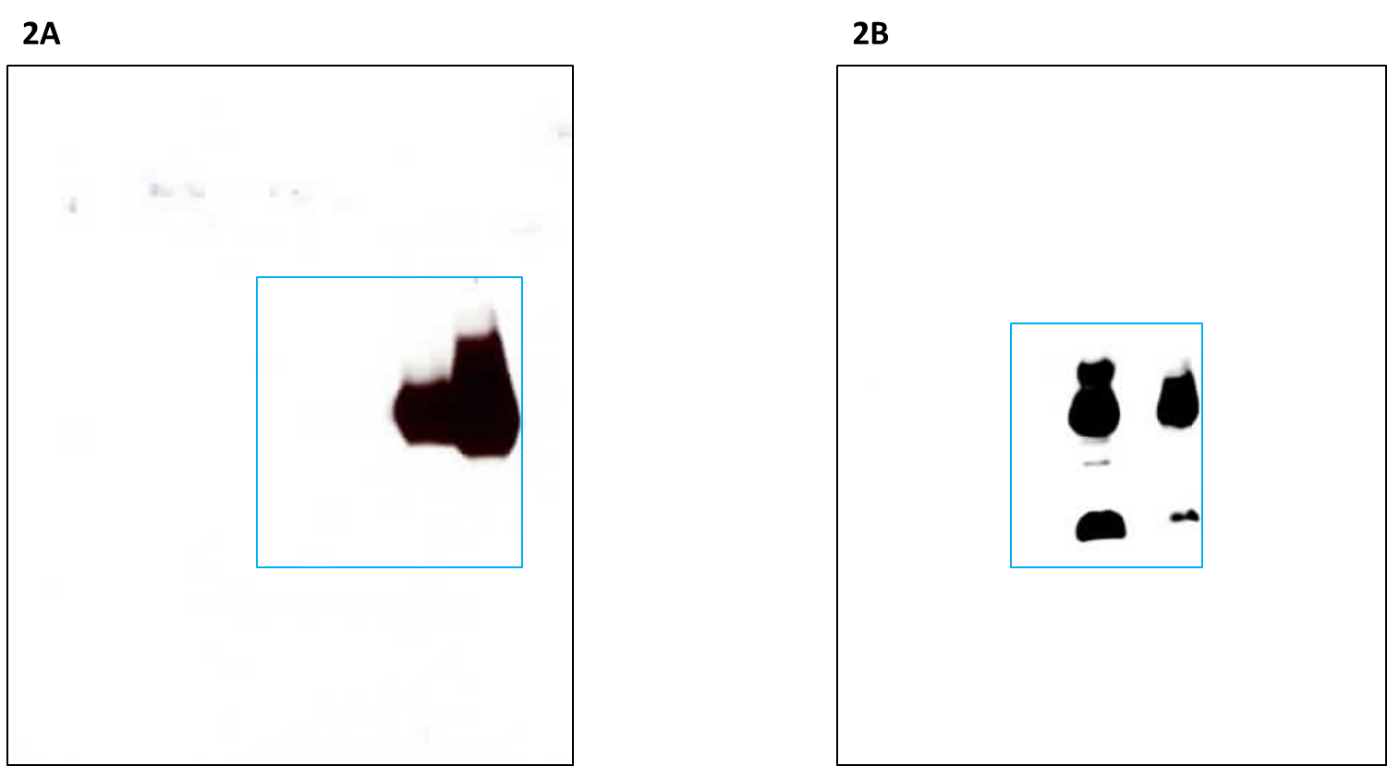


**Supplementary figure S1:** Full length of cropped versions of gels which were shown in figure 2 (A and B) in the main text. The full-length gels are shown in black boxes and cropped areas are shown in blue boxes.
